# Rationale and design of the Coronary Microvascular Angina Cardiac Magnetic Resonance Imaging (CorCMR) diagnostic study: the CorMicA CMR sub-study

**DOI:** 10.1136/openhrt-2018-000924

**Published:** 2018-12-30

**Authors:** David Corcoran, Thomas J Ford, Li-Yueh Hsu, Amedeo Chiribiri, Vanessa Orchard, Kenneth Mangion, Margaret McEntegart, Paul Rocchiccioli, Stuart Watkins, Richard Good, Katriona Brooksbank, Sandosh Padmanabhan, Naveed Sattar, Alex McConnachie, Keith G Oldroyd, Rhian M Touyz, Andrew Arai, Colin Berry

**Affiliations:** 1 British Heart Foundation Glasgow Cardiovascular Research Centre, University of Glasgow, Glasgow, UK; 2 West of Scotland Heart and Lung Centre, Golden Jubilee National Hospital, Glasgow, UK; 3 Advanced Cardiovascular Imaging Laboratory, National Heart, Lung, and Blood Institute, National Institutes of Health, Bethesda, Maryland, USA; 4 School of Biomedical Engineering and Imaging Sciences, Department of Cardiovascular Imaging, King’s College London, London, UK; 5 Robertson Centre for Biostatistics, University of Glasgow, Glasgow, UK

**Keywords:** angina, myocardial perfusion, cardiovascular magnetic resonance, coronary microvascular dysfunction, endoEndothelial dysfunction

## Abstract

**Introduction:**

Angina with no obstructive coronary artery disease (ANOCA) is a common syndrome with unmet clinical needs. Microvascular and vasospastic angina are relevant but may not be diagnosed without measuring coronary vascular function. The relationship between cardiovascular magnetic resonance (CMR)-derived myocardial blood flow (MBF) and reference invasive coronary function tests is uncertain. We hypothesise that multiparametric CMR assessment will be clinically useful in the ANOCA diagnostic pathway.

**Methods/analysis:**

The Stratified Medical Therapy Using Invasive Coronary Function Testing In Angina (CorMicA) trial is a prospective, blinded, randomised, sham-controlled study comparing two management approaches in patients with ANOCA. We aim to recruit consecutive patients with stable angina undergoing elective invasive coronary angiography. Eligible patients with ANOCA (n=150) will be randomised to invasive coronary artery function-guided diagnosis and treatment (intervention group) or not (control group). Based on these test results, patients will be stratified into disease endotypes: microvascular angina, vasospastic angina, mixed microvascular/vasospastic angina, obstructive epicardial coronary artery disease and non-cardiac chest pain. After randomisation in CorMicA, subjects will be invited to participate in the Coronary Microvascular Angina Cardiac Magnetic Resonance Imaging (CorCMR) substudy. Patients will undergo multiparametric CMR and have assessments of MBF (using a novel pixel-wise fully quantitative method), left ventricular function and mass, and tissue characterisation (T1 mapping and late gadolinium enhancement imaging). Abnormalities of myocardial perfusion and associations between MBF and invasive coronary artery function tests will be assessed. The CorCMR substudy represents the largest cohort of ANOCA patients with paired multiparametric CMR and comprehensive invasive coronary vascular function tests.

**Ethics/dissemination:**

The CorMicA trial and CorCMR substudy have UK REC approval (ref.16/WS/0192).

**Trial registration number:**

NCT03193294.

**Key questions**

**What is already known about this subject?**

- Angina with no obstructive coronary artery disease (ANOCA) is a common syndrome with unmet clinical needs.
- A significant proportion of these patients may suffer from microvascular and vasospastic angina.
- Diagnosis in this patients may be challending, are there are uncertain associations between the results of reference invasive diagnostic tests and the non-invasive ischaemia test results.

**What does this study add?**

- Novel CMR methods for measuring myocardial blood flow have not been validated in patients with ANOCA and underlying microvascular and vasospastic angina.
- The CorCMR substudy represents the largest cohort of ANOCA patients with paired multiparametric CMR and comprehensive invasive coronary vascular function tests. CorCMR will provide information on the diagnostic value of quantitative pixel-wise mapping of myocardial perfusion in patients with ANOCA.

**How might this impact on clinical practice?**

- In contrast to the reference standard invasive tests of coronary artery function, non-invasive imaging is safer and more acceptable to patients.
- The CorCMR sub-study presents a unique opportunity to assess and validate the diagnostic accuracy of fully-quantitative stress perfusion CMR in patients with ANOCA and comprehensive invasive coronary artery function testing.

## Introduction

### Angina and stable coronary syndromes (SCSs)

Ischaemic heart disease is the leading cause of mortality standardised by age and sex.^1^ In clinical practice, the diagnostic management of patients with angina pectoris focuses on the detection of obstructive epicardial coronary artery disease (CAD).^2^ In this stenosis-centred concept of myocardial ischaemia, angina is synonymous with obstructive CAD.^3^ There are well established treatment options for patients with epicardial CAD, namely optimal medical therapy and myocardial revascularisation by either percutaneous coronary intervention or coronary artery bypass grafting.^3^ However, the paradigm of angina pectoris resulting from obstructive epicardial CAD fails to account for the approximately one-third of patients who suffer from angina in whom obstructive CAD is excluded.^4^

Patients with angina and no obstructive coronary artery disease (ANOCA) present a diagnostic conundrum.^5^ The management of these patients is varied, and most patients fail to have a diagnosis made for the cause of their symptoms, receive no further diagnostic work-up, and have no therapeutic intervention. The underlying aetiology of chest pain symptoms and a ‘negative’ coronary angiogram is heterogeneous. However, a significant number of patients may have a disorder of coronary vascular function due to abnormal microvascular resistance or vasodilator capacity (coronary microvascular dysfunction (CMD)) or abnormal endothelial function (vasospastic disease).

The underlying pathogenesis in patients with ANOCA is unclear, as specific disease endotypes are not routinely tested for. CMD may result from coronary structural abnormalities, whereby decreased capillary luminal size and number result in increased microvascular resistance to myocardial blood flow (MBF) and reduced vasodilatory capacity.^6^ Functional abnormalities of the coronary epicardial vessels and microvasculature may result in either abnormal vasoconstriction or impaired vasodilatation, and these abnormalities may be secondary to either endothelium-dependent or endothelium-independent mechanisms.^7^

Abnormalities of coronary vascular function portend a worse prognosis in patients with both obstructive epicardial CAD and ANOCA.^8–12^ Therapeutic interventions are lacking in patients with ANOCA, and historical therapeutic studies have been performed in heterogeneous patient cohorts due to a lack of diagnostic tests to appropriately define endotypes of disease.^13–15^ There is a missing link between the use of diagnostic tests of coronary artery function, therapeutic agents with proven efficacy and health outcomes of patients with angina secondary to disorders of coronary vascular function. The term stable coronary syndrome (SCS) has been proposed to increase physician awareness of these conditions.^5^ CMD and vasospastic disease may result in ANOCA and myocardial ischaemia, and are recognised as a condition of unmet clinical need.^16^ The Coronary Vasomotion Disorders International Study Group (COVADIS) working group have proposed diagnostic criteria for disease endotypes in patients with ANOCA.^17^ COVADIS recommend a comprehensive testing strategy incorporating tests of coronary pressure, flow, resistance and endothelial function, in addition to the assessment of objective evidence of myocardial ischaemia.^17^

### Diagnostic testing in patients with ANOCA

Diagnosis of coronary microvascular and vasomotor dysfunction is challenging due to the heterogeneity of underlying disease mechanisms, the potentially patchy distribution of disease throughout the myocardium, and limited spatial resolution of existing diagnostic tests.^18 19^ There is no available in vivo technique for imaging the coronary microcirculation, and anatomical tests are fundamentally limited by their spatial resolution and the small size of the coronary microvasculature. Therefore, the diagnosis of microvascular angina and vasospastic angina is predominantly made with functional tests.

There is no accepted guideline-directed diagnostic algorithm for coronary vascular dysfunction in routine clinical practice.^17 20^ Invasive coronary angiography combined with adjunctive tests of coronary artery function represents the reference diagnostic approach for disorders of coronary vascular function. In contrast, non-invasive imaging involves less discomfort for patients, is safer than invasive procedures and is generally less expensive and more widely available. Recent developments with cardiovascular magnetic resonance (CMR) imaging now enable measurement of MBF with high spatial and temporal resolution.^21^ In addition, CMR permits the reference standard non-invasive assessment of left ventricular (LV) function and myocardial tissue characterisation.^22^

### Non-invasive ischaemia testing in ANOCA

The available non-invasive ischaemia tests were all validated for the detection of obstructive epicardial CAD. There has been a low yield of inducible myocardial ischaemia in ANOCA patients with traditional non-invasive ischaemia tests (e.g. exercise ECG testing, myocardial perfusion scintigraphy and stress echocardiography).^23^ However, non-invasive methods (namely stress perfusion positron emission tomography (PET) and CMR which image earlier in the ischaemic cascade and have greater spatial resolution) may provide new insights into the burden of myocardial ischaemia in patients with ANOCA. PET is the most studied modality for the assessment of myocardial perfusion and is considered to provide the reference standard assessment of MBF, but in real-world clinical practice, availability is limited due to cost. Conversely, CMR is more widely available and use is increasing.^24 25^

Coronary microvascular disease may be revealed by a deficit in MBF during stress CMR. The spatial distribution of this abnormality typically involves the subendocardium, which is the location of the microvascular plexus.^26^ In contrast, vasospastic angina occurs due to spontaneous spasm of the epicardial and microvascular vasculature. Vasospastic angina may not be detected by conventional stress testing that routinely use adenosine (an endothelial-independent vasodilator). The available data are conflicting on the role of perfusion CMR in patients with ANOCA.^26–28^ In contrast to qualitative analysis, semiquantitative perfusion methods have been investigated, and reduced myocardial perfusion reserve index (MPRi) has been found in patients with ANOCA.^29^ The incremental diagnostic value of fully quantitative perfusion CMR in patients with ANOCA is unknown.

### Associations between invasive and non-invasive diagnostic tests in patients with ANOCA

There are uncertain associations between the results of invasive diagnostic tests and the non-invasive ischaemia test results in patients with ANOCA. There is no accepted objective diagnostic threshold for the diagnosis of coronary vascular dysfunction (either abnormal myocardial flow or microvascular resistance), with either PET or perfusion CMR. Recent studies have investigated the relationships between invasive tests of coronary artery function and semiquantitative perfusion CMR analysis.^30 31^ Importantly, these patients have not undergone comprehensive coronary vascular function testing and in general moderate correlations were demonstrated (MPRi and index of microcirculatory resistance (IMR): r=−0.67; MPRi and coronary flow reserve (CFR): r=0.41).^30^

### The Stratified Medical Therapy Using Invasive Coronary Function Testing In Angina (CorMicA) trial

**Figure 1:**
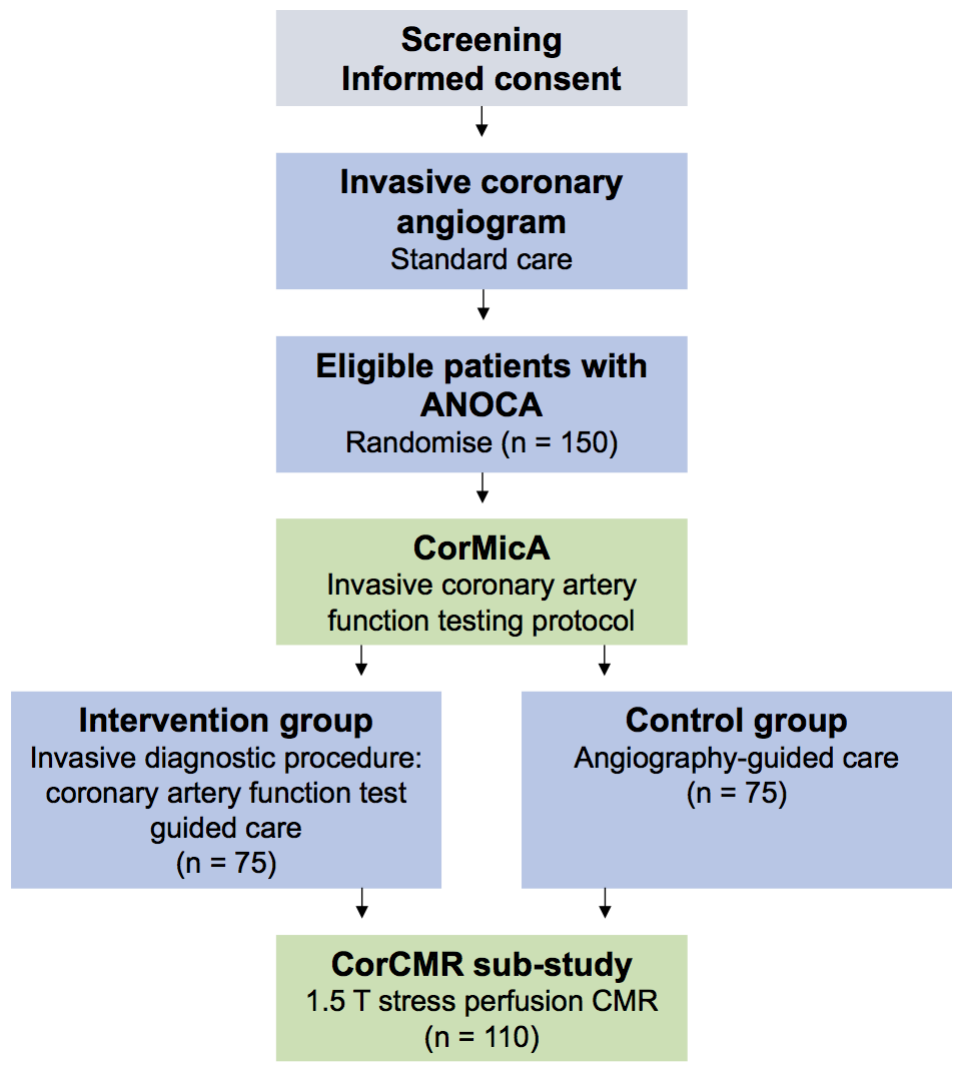
CorCMR substudy flow diagram. ANOCA, angina with no obstructive coronary artery disease; CMR, cardiovascular magnetic resonance; CorCMR, Coronary Microvascular Angina Cardiac MRI; CorMicA, coronary microvascular angina.

The Stratified Medical Therapy Using Invasive Coronary Function Testing In Angina (CorMicA) clinical trial is a proof-of-concept, prospective, blinded, randomised, sham-controlled study comparing two management approaches to the clinical problem of patients with ANOCA.^32^ CorMicA tests the hypothesis that stratified medicine guided by invasive coronary artery function testing (interventional diagnostic procedure (IDP)) in patients with ANOCA will facilitate diagnosis of the underlying disease endotype, direct therapeutic interventions aligned to the endotype and result in improved angina and well-being.^32^ Patients undergoing elective invasive coronary angiography for investigation of angina at two UK centres will be screened. Eligible patients with ANOCA (n=150) will be immediately randomised 1:1 to either coronary vascular function-guided diagnosis and treatment (intervention group/IDP disclosed) or not (control group/IDP sham procedure, results not disclosed) (figure 1).

The IDP consists of coronary artery function testing using a dual pressure-sensitive and temperature-sensitive guidewire and adenosine followed by intracoronary acetylcholine provocation testing. Assessment of microvascular resistance (IMR), microvascular vasodilatory capacity (resistance reserve ratio (RRR)), epicardial and microvascular vasodilatory capacity (CFR), endothelial function (acetylcholine provocation testing) and epicardial CAD (fractional flow reserve (FFR)) will be performed. Following these invasive tests of coronary artery function, patients will be classified into the following ANOCA disease endotypes (table 1): (1) microvascular angina; (2) vasospastic angina; (3) mixed microvascular angina and vasospastic angina; (4) obstructive epicardial CAD; and (5) non-cardiac chest pain. The disease endotypes are aligned with the COVADIS working group definitions.^17^

**Table 1:** Definitions of ANOCA disease endotypes

Disease endotype	Mechanism	Invasive diagnostic test
Microvascular angina	↑ Microvascular resistance	IMR ≥25
	↓ Coronary vasorelaxation	CFR <2.0
	↓ Microvascular vasodilator capacity	RRR <2.0
	Microvascular spasm	ACh testing: angina, ischaemic ST segment deviation, epicardial coronary vasoconstriction <90%.
Vasospastic angina	Epicardial spasm	ACh testing: angina, ischaemic ST segment deviation, >90% epicardial coronary vasoconstriction.
Mixed microvascular and vasospastic angina	CMD and epicardial vasospasm	Epicardial vasospasm and either ↑ microvascular resistance, ↓ coronary vasorelaxation or ↓ microvascular vasodilator capacity.
Obstructive epicardial CAD	Epicardial stenosis	>50% lesion by diameter stenosis in epicardial artery >2.5 mm or FFR ≤0.80.
Non-cardiac pain	Nil	Exclusion of epicardial (FFR >0.8), microvascular (CFR >2.0, IMR <25, RRR >2.0) vasospasm (normal ACh response).

ACh, acetylcholine; ANOCA, angina with no obstructive coronary artery disease; CAD, coronary artery disease; CFR, coronary flow reserve; CMD, coronary microvascular dysfunction; FFR, fractional flow reserve; IMR, index of microcirculatory resistance; RRR, relative resistance ratio.

Patients in the control group will receive standard care based on the interpretation of the invasive coronary angiogram alone. Patients in the intervention group will have care based on the disease endotypes disclosed by invasive tests of artery function, and pharmacotherapy linked to the underlying disease endotype will be commenced.^32^ In each case, the diagnosis (endotype) will be assessed by the attending cardiologist before and after the coronary angiogram. In the intervention group, the diagnosis is re-evaluated after disclosure of the coronary function test results at the end of the invasive procedure. The primary outcome is the mean difference in the within-subject change in Seattle Angina Questionnaire score between the groups at 6 months from baseline. A prespecified substudy of the CorMicA trial investigated the frequency of peripheral microvascular dysfunction in patients with ANOCA.^33^

### Coronary Microvascular Angina Cardiac Magnetic Resonance Imaging (CorCMR) sub-study

Novel CMR methods for measuring MBF have not been validated in patients with ANOCA and underlying microvascular and vasospastic angina. The CorCMR substudy of the CorMicA clinical trial presents a unique opportunity to assess and validate the diagnostic accuracy of fully quantitative stress perfusion CMR in patients with ANOCA and comprehensive invasive coronary artery function testing.

### Hypothesis

We hypothesise that abnormal myocardial perfusion, as revealed by a novel fully quantitative pixel-wise CMR perfusion sequence, will be prevalent in a contemporary UK cohort of patients presenting with ANOCA and that multiparametric CMR imaging will be clinically useful in the diagnostic pathway of patients with ANOCA.

### Aims

We aim to assess the diagnostic validity of quantitative stress perfusion CMR in patients with ANOCA and specific disease endotypes of coronary vascular dysfunction. Our specific aims are to assess:

1. The proportion of ANOCA patients with abnormal myocardial perfusion. There is no accepted threshold for abnormal MPR. Thresholds for reduced CFR of 1.5–2.6 have been described, however <2.0 is commonly used.^10 34–38^ We will therefore assess the concordance of patient classification using this MPR <2.0 threshold against endotypes determined by invasive testing. Second, we will assess the MPR value with the highest area under the curve (AUC) for classification of microvascular angina based on the invasive tests (reference classification). Third, we will assess the proportion of ANOCA endotypes with other abnormal perfusion metrics (reduced stress MBF, abnormal endocardial:epicardial MBF ratio and abnormal myocardial dyssynchrony index).^39^
2. The diagnostic accuracy of the perfusion CMR metrics for abnormal invasive tests of coronary artery function (IMR >25, CFR <2.0 and RRR <2.0) and the associations between specific disease endotypes and myocardial perfusion in patients with ANOCA. We also aim to assess the correlation between abnormal CMR-derived perfusion and invasive tests of coronary artery function, of qualitative versus quantitative perfusion methods.
3. The proportion of patients with ANOCA and abnormal myocardial tissue characterisation derived from CMR (as revealed by T1 parametric mapping and late gadolinium enhancement (LGE) imaging) and its association with abnormal myocardial perfusion.
4. The associations between baseline patient characteristics and abnormal myocardial perfusion.
5. The proportion of patients with a change in diagnosis based on quantitative CMR findings, as compared with the initial diagnosis by the attending cardiologist based on the coronary angiogram.

## Methods and analysis

### Study design

Prespecified substudy of the CorMicA stratified medical therapy clinical trial.

### Setting

Patients referred from 14 acute hospitals to two large regional UK hospitals (Golden Jubilee National Hospital and Hairmyres Hospital) providing invasive care to all patients in the West of Scotland (population 2.5 million). All CMR studies will be performed at the Golden Jubilee National Hospital.

### Participants

Consecutive outpatients undergoing clinically indicated elective diagnostic angiography for investigation of suspected angina will be screened and invited to participate in the CorMicA trial. Informed consent is obtained before the invasive coronary angiogram. A minimum of 400 consecutive patients undergoing elective invasive coronary angiography is expected to be screened to enrol 150 subjects with ANOCA within 24 months. Consenting patients who are not randomised (e.g. demonstrated to have obstructive epicardial disease or logistical reasons) will enter a registry. Only patients randomised in the CorMicA trial (both in the intervention and control groups) are eligible to participate in the CorCMR substudy.

#### Inclusion criteria

Age ≥18 years, a clinically indicated plan for invasive coronary angiography and symptoms of angina (according to the Rose angina questionnaire).

#### Exclusion criteria

A non-coronary indication for invasive angiography (e.g. valvular heart disease and cardiomyopathy), obstructive epicardial CAD disease evident in a main coronary artery (diameter >2.5 mm) (defined as diameter stenosis >50% or FFR ≤0.80) and contraindication to contrast-enhanced CMR (glomerular filtration rate <30 mL/min, CMR unsafe devices).

### CMR protocol and analysis

**Figure 2:**
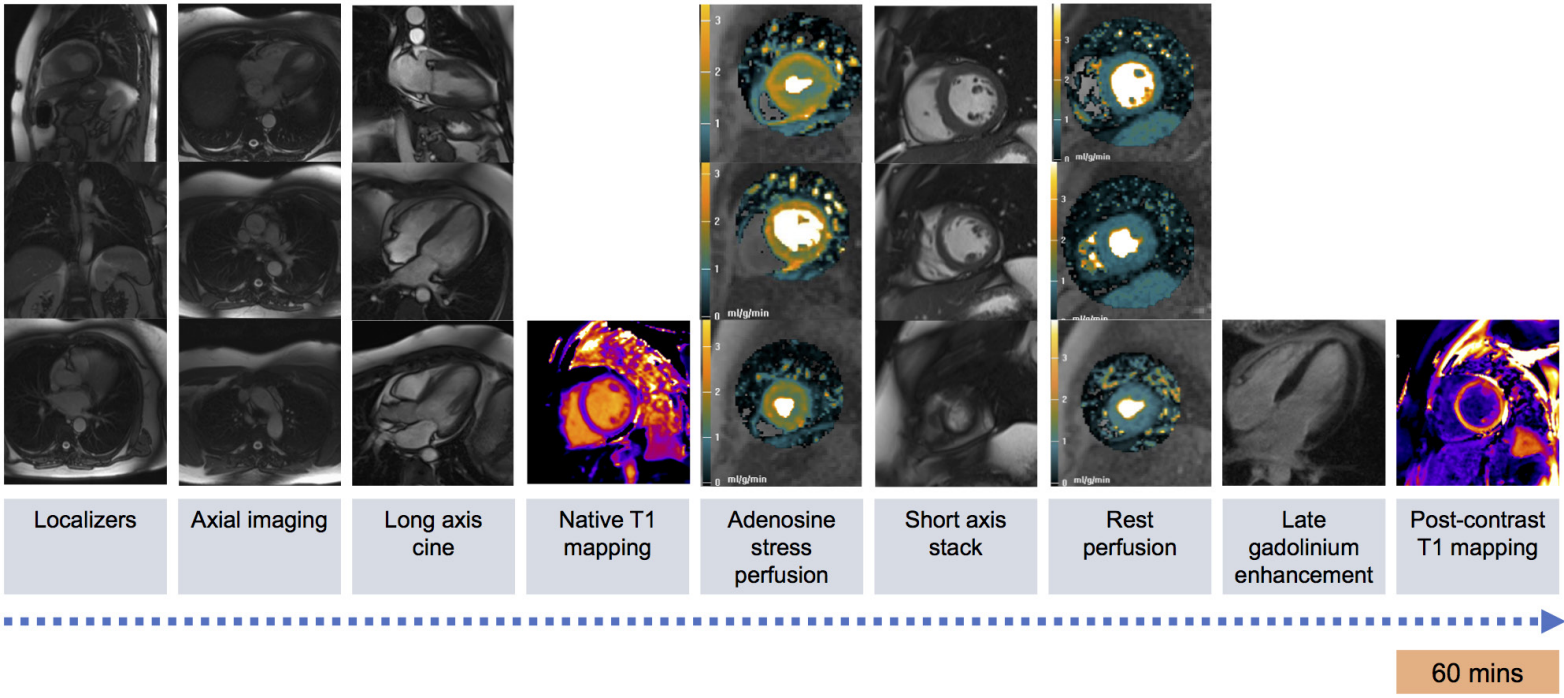
CorCMR multiparametric imaging protocol. CorCMR, Coronary Microvascular Angina Cardiac MRI.

Patients will undergo perfusion CMR within 42 days of the CorMicA trial invasive coronary artery function testing. CMR studies will be performed at 1.5 Tesla (Siemens MAGNETOM Avanto, Erlangen, Germany), and patients will undergo a standardised CMR protocol. All patients will be asked to abstain from caffeine-containing beverages or foodstuffs for 24 hours and vasoactive medications for 48 hours prior to the CMR examination. All scan acquisitions will be spatially coregistered. All CMR analyses will be performed by analysts blinded to the invasive coronary artery function test results. The standardised CMR protocol is demonstrated in figure 2.

#### Myocardial perfusion

Stress and rest first-pass perfusion imaging will be performed using an echo planar imaging dual-sequence investigational perfusion method, which consists of a low resolution arterial input function image, followed by three short axis (base, mid and apex) myocardial images during each R–R interval.^40–42^ Vasodilator stress will be achieved with adenosine infusion 140–210 µg/kg/min for 3 min. Resting first-pass perfusion will be performed at least 10 min later.

The raw stress and rest perfusion images will be qualitatively assessed for inducible or fixed perfusion defects. Perfusion defects will be reported on a segmental basis according to the American Heart Association 16-segment model.^43^ Dark banding artefact will be adjudicated based on standardised criteria.^22^

Pixel-wise perfusion maps will be generated and analysed to derive fully quantitative MBF estimates on a pixel-wise basis in mL/g/min of myocardium. The pixel-wise perfusion method uses a series of automated postprocessing steps on the raw Digital Imaging and Communications in Medicine images to generate fully quantitative pixel maps.^21^ The pixel-wise time-signal intensity curves will then be quantified using model-constrained Fermi deconvolution.^44–46^

The myocardial perfusion dyssynchrony index is a novel perfusion metric that assesses temporal differences in the distribution of gadolinium-based contrast media myocardial wash-in. This index has the potential to increase the diagnostic sensitivity and specificity of perfusion CMR for abnormalities of myocardial perfusion in patients with ANOCA.^39^ Global perfusion dyssynchrony indices will be measured as both variance [s^2^] and percentage coefficient of variation of time to maximum signal intensity upslope and time to peak myocardial signal intensity enhancement.

#### Extracardiac anatomy and LV volumes, function and mass

Fast gradient echo images in the axial, coronal and sagittal planes will be qualitatively assessed for extra cardiac anatomy and pathology and clinically relevant incidental findings.

Steady-state free procession ‘cine’ imaging using a trueFISP sequence (multislice single-shot breath-hold true fast imaging) will be performed in the three long-axis and short-axis planes for assessment of LV volumes, function and mass.

#### Myocardial tissue characterisation

Native T1 mapping will be performed using a modified look-locker inversion recovery investigational prototype sequence. Images will be obtained in three short-axis images (base, mid and apex). T1 mapping will be performed pre- and post-gadolinium contrast to assess the myocardial native T1 relaxation time and estimate the myocardial extracellular volume (ECV) in both the mid-septum and globally.^47^ For the calculation of ECV, blood haematocrit will be measured at baseline on enrolment into the CorMicA trial. ECV mapping analysis will be performed offline using proprietary software.

LGE imaging will be performed using a segmented phase-sensitive inversion recovery turbo fast low-angle shot imaging sequence.^48^ Images will be obtained in the three long-axis planes and short-axis images covering the entire LV myocardium. The pattern and burden of hyperenhancement will be qualitatively and quantitatively assessed.

### Primary and secondary outcomes

#### Primary outcome

Abnormal myocardial perfusion reserve (global MPR <2.0).

#### Secondary outcomes

A summary of the secondary outcomes is shown in Box 1.

Box 2. **CorCMR substudy secondary outcomes and analyses**

**LV volumes, function and mass**

- LV EF
- LVEDV
- LV EDV index
- LV ESV
- LV ESV index
- LV mass
- LV mass index
- LV CO
- LV CO index

**Atrial area**

- Left atrial area (cm^2^)
- Left atria dilated (Y/N)
- Right atrial area (cm^2^)
- Right atria dilated (Y/N)

**Qualitative perfusion analysis**

- Abnormal perfusion (Y/N)
- Number of abnormal segments (n)
- Transmurality of perfusion defects (%)
- Pattern (epicardial, microvascular, mixed and equivocal)

**Quantitative perfusion analysis**

- Global MPR <2.0 (Y/N)
- Segmental/AHA MPR
- Global stress and rest MBF
- Segmental/AHA territory stress and rest MBF
- Global stress endocardial:epicardial MPR ratio
- Segmental/AHA territory endocardial:epicardial MPR ratio

**Perfusion dyssynchrony analysis**

- Myocardial perfusion dyssynchrony index (variance and coefficient of variation of the time to maximum signal intensity upslope and time to peak myocardial signal intensity enhancement)

**Adenosine vasodilator stress response**

- Splenic switch-off (Y/N)
- HR and BPchange
- Rate-pressure product at rest and stress

**Late gadolinium enhancement imaging**

- Abnormal LGE (Y/N).
- Number of affected AHA segments (n)
- Pattern of abnormal LGE (ischaemic and non-ischaemic)
- Myocardial infarct scar burden (transmurality, infarct mass, infarct mass as percentage LV mass)

**Native T1 and ECV mapping**

- Native T1 (global and midseptal values)
- ECV (global and midseptal values)

**Myocardial strain**

- Feature-tracking and DENSE (two methods)
- Longitudinal strain (global and by AHA segment)
- Circumferential strain (global and by AHA segment)
- Radial strain (global and by AHA segment)

**Incidental findings**

- Present (Y/N)
- Clinically significant (Y/N)

**Associations of abnormal perfusion with baseline characteristics**

- Sex, age and traditional CAD risk factors
- CAD risk scores (JBS3 and ASSIGN)
- Gensini score of epicardial plaque burden

**Associations of abnormal perfusion with invasive coronary artery function testing data**

- Association of myocardial perfusion with IMR, CFR, RRR and endothelial function testing (continuous and binary)
- Subset of patients with multivessel invasive coronary artery function measurements
- Association with clinical diagnosis (ANOCA disease endotype)

**Associations of abnormal perfusion with baseline CMR data**

- LV volumes and mass
- Myocardial strain
- Native T1 relaxation time and ECV
- Presence of LGE

**Diagnostic accuracy**

- Diagnostic accuracy of quantitative perfusion CMR to detect abnormal IMR, CFR, RRR and endothelial dysfunction

AHA, American Heart Association; ANOCA, angina with no obstructive coronary artery disease; BP, blood pressure; CFR, coronary flow reserve; CAD, coronary artery disease; CO, cardiac output; CorCMR, Coronary Microvascular Angina Cardiac MRI; EF, ejection fraction; EDV, end-diastolic volume; ESV, end-systolic volume; ECV, extracellular volume; HR, heart rate; IMR, index of microcirculatory resistance; LV, left ventricular; LGE, late gadolinium enhancement; MBF, myocardial blood flow; MPR, myocardial perfusion reserve; RRR, relative resistance ratio,

### Statistical analyses

The CorMicA trial has a comprehensive statistical analysis plan that governs all statistical aspects of the study authored by the trial statistician. The statistical analysis plan includes the prespecified CorCMR substudy that is designed to assess for associations between CMR measures and invasive measures and endotypes (reference dataset). Continuous outcomes will be analysed using linear regression with adjustment for baseline levels where available. Where continuous data are clearly not normally distributed, standard transformations will be applied to achieve approximate normality prior to analysis. Appropriate alternative regression methods will be applied to other types of data (eg, logistic regression for binary outcomes).

#### Sample size calculation

The primary outcome is the proportion of patients with an MPR<2.0. The proportion of patients with microvascular angina defined by invasive endotyping will be assessed. We will further assess the MPR ratio with the highest AUC for microvascular angina classified invasively.

Considering the correlation between CFR measured invasively and MPR measured non-invasively within the common territory of a major epicardial coronary artery, then a sample size of 110 subjects would enable a minimum clinically significant correlation of 0.3 to be detected with 90% power at a 5% significance level. If only 60 subjects have available data, then 80% power would be available to detect a correlation of 0.37 at the 5% level.

## Discussion

The British Heart Foundation CorMicA trial will assess a routine stratified medicine strategy in a large cohort of prospectively enrolled patients with ANOCA. The prespecified CorCMR substudy will involve comprehensive invasive tests of coronary artery function paired with multiparametric perfusion CMR studies. The analysis will provide information on the diagnostic value of quantitative pixel-wise mapping of myocardial perfusion in this population.

Contemporary guidelines recommend functional testing, including with CMR, to assess for myocardial ischaemia in patients in whom multidetector CT coronary angiography has shown CAD of uncertain functional significance or is non-diagnostic.^49^ Increasingly, patients are referred to the catheter laboratory based on the results of anatomical imaging using CT coronary angiography or with no prior tests based on symptoms and a high likelihood of CAD.^49 50^ The CorCMR substudy in CorMicA aims to inform this gap.

The CorCMR substudy will be performed on a 1.5 Tesla MRI scanner. In comparison, 3.0 Tesla imaging permits improved signal-to-noise ratio and provides higher in-plane spatial resolution perfusion imaging.^51 52^ However, CMR imaging in the NHS is most widely performed at 1.5 Tesla, hence the results from CorCMR are clinically relevant and transferable.

### Literature review

Patients with confirmed microvascular or vasospastic angina have a precise diagnosis of the underlying disease endotype, and pharmacotherapy may be commenced as appropriate. Conversely, patients with normal invasive coronary artery function tests may have antianginal therapy appropriately discontinued, and alternative causes of chest pain investigated. In comparison with invasive tests of coronary artery function, non-invasive CMR imaging may be more attractive to patients, but at present, the role of CMR in the diagnostic work-up of patients is uncertain. CorCMR will provide data on the role of CMR in the diagnosis of patients with ANOCA. Traditional non-invasive ischaemia testing in patients with ANOCA has provided mixed results. Panza *et al*^23^ investigated the yield of non-invasive ischaemia testing in 70 patients with ANOCA. Patients underwent exercise ECG testing, radionuclide angiography, myocardial perfusion scintigraphy and dobutamine stress echocardiography. Abnormal test results were detected in 31%, 16%, 18% and 0% respectively, with no concordance between the test results. This led to the conclusion that patients with ANOCA did not have inducible myocardial ischaemia as a cause for their symptoms. However, there is evidence that perfusion CMR may detect abnormalities of MBF in patients with ANOCA. Panting *et al*^26^ first described the use of adenosine stress perfusion CMR in patients with ANOCA. Twenty patients with syndrome X and 10 control subjects were included, and subendocardial hypoperfusion was demonstrated in patients with syndrome X using a semiquantitative perfusion metric. A subset of 118 women with ANOCA enrolled in the National Heart, Lung and Blood Institute-sponsored multicentre Women’s Ischemic Syndrome Evaluation (WISE) study underwent stress perfusion CMR and invasive microvascular function testing with Doppler wire-derived CFR and endothelial function testing with intracoronary acetylcholine.^29^ Stress perfusion CMR was also performed in 21 asymptomatic control subjects. Reduced global MPRi was found in patients with ANOCA compared with controls (1.79 vs 2.23, p<0.0001), and a lower MPRi was predictive of ≥1 abnormal invasive coronary vascular function metric (OR=0.78, p<0.0001). An MPRi threshold of 1.84 predicted an abnormal invasive coronary function metric with sensitivity of 73% and specificity of 74%. Qualitative analysis of the perfusion studies found no significant differences between the study and control groups (summed segments with abnormal perfusion 6.66 vs. 4.45, p=0.09), suggesting that quantitative perfusion analyses may have greater sensitivity for abnormal perfusion in patients with ANOCA.

CorCMR will inform the nascent evidence on the presence and magnitude of associations between invasive and non-invasive assessments of coronary vascular function. Liu *et al* compared semiquantitative perfusion CMR against IMR measurement in 50 patients with ANOCA and 20 age-matched healthy control subjects.^30^ In a ROC analysis, an MPRi threshold of 1.4 was optimal for the detection of myocardium with inducible ischaemia in patients with obstructive epicardial CAD (FFR ≤0.8) and in patients with ANOCA. Similarly, Williams *et al*^31^ performed semiquantitative perfusion CMR and measured thermodilution and Doppler flow wire-derived metrics of microvascular resistance (IMR and hyperaemic microvascular resistance (hMR), respectively) in a heterogeneous cohort of 54 patients (44 with acute myocardial infarction and 10 with stable angina). Microvascular function testing was performed in unobstructed epicardial coronary arteries (FFR >0.8). MPRi correlated with hMR (r=0.58, p<0.001) but not IMR (r=−0.27, p=0.15). These comparisons are between a non-invasive metric of vasodilatory capacity (MPRi) and an invasive metric of microvascular resistance (IMR and hMR), which may more specifically reflect fixed structural changes in the microcirculation (rather than vasodilator capacity).

CMR permits the reference-standard non-invasive assessment of myocardial tissue characterisation. CorCMR will provide data on diffuse interstitial fibrosis, ECV and myocardial scar. The role of T1 mapping and ECV analysis in patients with ANOCA is uncertain. In a substudy of the iPOWER natural history study, 54 women with ANOCA underwent native T1 mapping and ECV analysis, PET-derived MBF measurement, and transthoracic Doppler echocardiography CFR assessment.^53^ There was no correlation found between abnormal coronary vascular function as revealed by PET or echocardiography-derived CFR, and myocardial native T1 or ECV. Occult myocardial infarction in patients with ANOCA may be clinically relevant. In a substudy of the WISE cohort, Wei *et al*^54^ performed CMR imaging in 340 female patients with ANOCA. A total of 26 patients (8%) had hyperenhancement on LGE imaging, with 18 patients having evidence of occult myocardial infarction (mean scar size 5.1 g), and 8 patients had a non-ischaemic pattern (mean scar size 8.9 g).

## Ethics and dissemination

Progress in the trial will be monitored by the trial anager (KB) and sponsor. The study will be subject to internal and external audit that is routinely coordinated by the sponsor. An annual report will be submitted to the Research Ethics Committee on a 12-month basis. The flow diagram illustrates conservative estimates of patient enrolment and activity on a single site. The study will follow Standards for Reporting of Diagnostic Accuracy (STARD) (http://www.equator-network.org/reporting-guidelines/stard/) and Consolidated Standards of Reporting Trials (http://www.consort-statement.org/) guidelines.

The CorCMR data will be presented at conferences and/or published in peer-reviewed journals.
